# Management of Neonatal Hepatic Hemangiomas: A Single-Center Experience Focused on Challenging Cases

**DOI:** 10.3390/jcm13102839

**Published:** 2024-05-11

**Authors:** Sumin Lee, Hojong Jeon, Jungho Han, In-Kyu Song, Seung Hwan Baek, Sungbo Shim, Hoseon Eun, Min Soo Park, Hyeonguk Jang, Jeong Eun Shin, Kyong Ihn

**Affiliations:** 1 Division of Neonatology, Department of Pediatrics, Severance Children’s Hospital, Yonsei University College of Medicine, Seoul 03722, Republic of Korea; sum52@yuhs.ac (S.L.); feagd@yuhs.ac (J.H.); igsong@yuhs.ac (I.-K.S.); baekga@yuhs.ac (S.H.B.); sbshim10@yuhs.ac (S.S.); hseun@yuhs.ac (H.E.); minspark@yuhs.ac (M.S.P.); 2 Division of Pediatric Surgery, Department of Surgery, National Health Insurance Service Ilsan Hospital, Goyang-si 10444, Republic of Korea; gdijhj@nhimc.or.kr; 3 Department of Pathology, Severance Hospital, Yonsei University College of Medicine, Seoul 03722, Republic of Korea; 4 Division of Pediatric Surgery, Department of Surgery, Severance Children’s Hospital, Yonsei University College of Medicine, Seoul 03722, Republic of Korea

**Keywords:** hepatic hemangioma, alpha-fetoprotein, propranolol, sirolimus, treatment outcome

## Abstract

**Background**: Management of hepatic hemangioma (HH) in infancy ranges from close monitoring to surgical resection. We analyzed the clinical characteristics and outcomes of HH according to its treatment options, with particular focus on challenging cases. **Methods**: Data of patients diagnosed with HHs in their first year of life and followed up for at least 1 year were retrospectively reviewed and divided into treatment and observation groups. Serial imaging results, serum alpha-fetoprotein (AFP) levels, medications, and clinical outcomes were compared. The detailed clinical progress in the treatment group was reviewed separately. **Results**: A total of 87 patients (75 in the observation group and 12 in the treatment group) were included. The median HH size at the initial diagnosis and the maximum size were significantly larger in the treatment group than the observation group (2.2 [0.5–10.3] cm vs. 1.0 [0.4–4.0] cm and 2.1 [0.7–13.2] vs. 1.1 [0.4–4.0], respectively; all *p* < 0.05]. The median initial and last serum AFP levels were significantly higher in the treatment group than in the observation group (76,818.7 vs. 627.2 and 98.4 vs. 8.7, respectively; all *p* < 0.05). Serum AFP levels in both groups rapidly declined during the first 3 months of life and were almost undetectable after 6 months. Among the challenging cases, a large (14 × 10 × 6.5 cm sized) focal HH was successfully treated using stepwise medical-to-surgical treatment. **Conclusions**: Patients with large HH and mild symptoms can be treated using stepwise pharmacotherapy. More aggressive surgical treatment of tumors unresponsive to initial pharmacotherapy may help shorten the treatment period and improve outcomes.

## 1. Introduction

Approximately 8.6% of all pediatric liver tumors, 60% of which are hepatic hemangiomas (HHs), are reported to occur before the age of 2 months [1]. HHs are classified into two types: congenital HH (CH), which is detected prenatally and reaches its maximum size at birth, and infantile HH (IH), which is detected in infancy and continues to grow until 6 to 12 months of age, usually involuting by 3–9 years of age [2,3]. Both types of HH have various clinical manifestations, are sometimes asymptomatic, and are associated with life-threatening complications, such as respiratory difficulty, congestive heart failure, abdominal compartment syndrome, and hematological abnormalities [3,4].

The diagnosis of HH is made with the clinical presentation and radiologic imaging [2]. Histologic confirmation is rarely required, but may be indicated in an atypical clinical and imaging presentation to exclude malignancy [2,3]. The radiologic features specific to HH are well defined, and Doppler ultrasound of the liver is usually sufficient to make a diagnosis at the initial presentation. If the diagnosis is unclear, magnetic resonance imaging (MRI) with contrast can be considered [5]. Serum alpha-fetoprotein (AFP) levels play an important role in distinguishing HH from malignant liver tumors such as hepatoblastoma. Although elevated serum AFP levels are common during the proliferative phase of HHs, a rising trend in AFP levels during follow-up warrants the suspicion of malignant liver tumors [2,5]. The treatment modalities of HH are variable including observation, pharmacological therapy, radiological intervention, and surgical resection. Selecting the optimal approach relies on tumor characteristics, as well as the severity of clinical symptoms, highlighting the importance of an early diagnosis and timely management, especially in cases with life-threatening complications [2].

The American Society of Pediatric Hematology Oncology Vascular Anomalies Special Interest Group recently suggested clinical guidelines for the diagnosis, screening, and monitoring of HHs [5]. Although histological markers (Glut-1) and the history of the in utero presentation of hepatic masses are key differences between CH and IH, the screening and monitoring strategies for both types of HH are similar [5,6]. Moreover, decision-making regarding whether to treat or observe HH primarily depends on the presence of complications, regardless of the HH subtype. However, as there are no common guidelines for the management of HH to date, the final decision regarding when and how to treat HH is based on the judgment of the physician [3,5].

In this study, we aimed to analyze real-world clinical data of patients with HH, including their serial imaging and laboratory results, and present the differences in their clinical outcomes according to the treatments they received. In addition, we reviewed three challenging cases with successful treatment outcomes with the aim of suggesting tailored treatment options for patients with HH based on our 15-year experience at a tertiary center in Korea. 

## 2. Materials and Methods

### 2.1. Study Population and Clinical Characteristics

The study protocol was approved by the Institutional Review Board of Severance Hospital (approval no.: 4-2023-0249; date of approval: 15 March 2024). Patients diagnosed with HH in the first year of life at Severance Children’s Hospital between 2005 and 2023 were screened, and those who were followed up for at least 1 year or showed a complete resolution of the mass were included in this study. Patients diagnosed after 1 year of age and patients with a single ultrasound examination and no further follow-up were excluded. Data on perinatal characteristics (gestational age, birth weight, sex, and antenatal suspicion of HH) and postnatal clinical findings (type of HH; symptoms related to HH; findings of serial imaging studies including ultrasonography (US), computed tomography (CT), and MRI; laboratory results including AFP level; and clinical outcomes) were extracted from medical records and reviewed. The patients were divided into two groups based on whether they received therapeutic interventions: a treatment group and an observation group. Treatment protocols were followed at the discretion of each physician. 

### 2.2. Diagnosis and Measurement of HH

Age at the HH diagnosis was defined as the postnatal day of the initial detection of the lesion on imaging studies (US, MRI, or CT), regardless of the purpose of the imaging study. Lesions were classified as “focal”, “multifocal”, or “diffuse” according to the radiological classification suggested by Christison-Legay et al. [7]. HH size was defined as the diameter of the largest lesion on US.

### 2.3. Measurement of Half-Life of Serum Alpha-Fetoprotein

All serum AFP levels measured during the follow-up period were recorded. Half-life of AFP was calculated using the method described in previous research: half-life (T) = Δt/Log_2_ (M_0_/M_1_), where M_0_ is the initial AFP level and M_1_ is the value obtained at each timepoint during follow-up [8]. Data from patients who underwent surgical treatment were excluded from the calculation of AFP half-life.

### 2.4. Treatment Indications, Options, and Outcomes

All medical treatments, radiological interventions with embolization, and surgical resections were included as treatment options. Oral propranolol (1 mg/kg/day to 3 mg/kg/day), oral prednisolone (1 mg/kg/day to 2 mg/kg/day), oral sirolimus (1.6 mg/m^2^/day), and subcutaneous interferon alpha (3 million units/m^2^/day) were used as medical treatment. Indications for treatment were classified into four categories: (1) large HH that induced life-threatening symptoms, (2) HHs that increased in number and size such that spontaneous regression was not expected, (3) diffuse HHs accompanied by hepatic failure, and (4) involvement of other organs, including the skin. Response to treatment was defined as complete resolution (not visible on US), partial resolution (visible on US but decrease in size and number), or no resolution (similar or increase in size and number). 

### 2.5. Study Outcomes and Statistical Analysis

The primary outcome of this study was the maximum size of HH. Secondary outcomes were the treatment response and change in serum AFP levels. Individual changes in HH size and AFP levels were plotted using descriptive statistics. The comparison of clinical characteristics and progress between the treatment and observation groups was performed using Fisher’s exact test for categorical variables. Continuous variables were tested for normality using the Kolmogorov–Smirnov test, and since most variables were not normally distributed, the Mann–Whitney *U* test was used. Linear regression analyses were performed to analyze AFP half-life and age. Statistical significance was set at *p* < 0.05. All data were expressed as a number (percentage of each group) or the median (range). Statistical analyses were performed using SPSS version 27 (IBM Co., Armonk, NY, USA). 

## 3. Results

### 3.1. Demographic Characteristics and Clinical Outcomes of the Study Population

A total of 87 patients (75 in the observation group and 12 in the treatment group) were analyzed in this study. Regarding the timing of the diagnosis, 14.9% (*n* = 13) cases were diagnosed prenatally. The median (range) age at the diagnosis was 61 (21–126) days, and the age of the patients in the treatment group was significantly younger than that of patients in the observation group (15.5 days vs. 75 days, *p* = 0.002). Regarding HH appearance, most of the lesions were focal (treatment vs. observation groups, 58.3% vs. 72%). Two patients had “diffuse” HH and both underwent treatment. The median (range) HH size at the initial diagnosis and the median maximum size were significantly larger in the treatment group than in the observation group (2.2 [0.5–10.3] cm vs. 1.0 [0.4–4.0] cm; *p* = 0.035, and 2.1 [0.7–13.2] vs. 1.1 [0.4–4.0]; *p* = 0.007). Three patients (25.0%) in the treatment group had heart failure. The median initial and last serum AFP levels were significantly higher in the treatment group than in the observation group (76,818.7 vs. 627.2; *p* = 0.001 and 98.4 vs. 8.7; *p* = 0.026). In both treatment and observation groups, the majority of the patients had a complete resolution of HHs followed by partial resolution and no response (60% vs. 66.7%; 25% vs. 28%; 8.3% vs. 12%, *p* = 1.000) The median (range) duration of the follow-up was 581 (224–946) days, and there was no difference between the two groups. The clinical characteristics of the study population are summarized in Table 1.

### 3.2. Individual Clinical Courses and Outcomes of the Treatment Group

The detailed information of the 12 patients in the treatment group is presented in Table 2. Of the 12 patients, 4 (33.3%) were diagnosed prenatally. Focal, multifocal, and diffuse HHs were diagnosed in seven (58.3%), three (25%), and two (16.7%) patients, respectively. Eight (66.7%) patients exhibited clinical symptoms associated with HH, including congestive heart failure, hypothyroidism, feeding difficulty, and respiratory difficulties. Four (33.3%) patients were asymptomatic but underwent treatment because an increase in the sizes or number of the masses was observed. Patient 7, who presented with a 5.8 cm sized focal HH and mild feeding difficulty, was successfully treated using oral beta-blockers. Patient 10 presented with a 7.4 cm sized focal HH, accompanied by feeding difficulty and abdominal distension. The patient was treated using beta-blockers; however, the mass continued to increase in size up to 7.4 cm. The subsequent inclusion of oral prednisolone and sirolimus in the treatment regimen led to reduction in the size of the mass and the resolution of the associated symptoms. Among the three patients who had heart failure, two underwent a surgical resection of the mass (Patients 1 and 11) and ultimately achieved complete resolution, whereas the remaining patient, who had diffuse HH, did not survive (Patient 3). 

### 3.3. Trends in HH Size in the First 2 Years of Life

**Figure 1:**
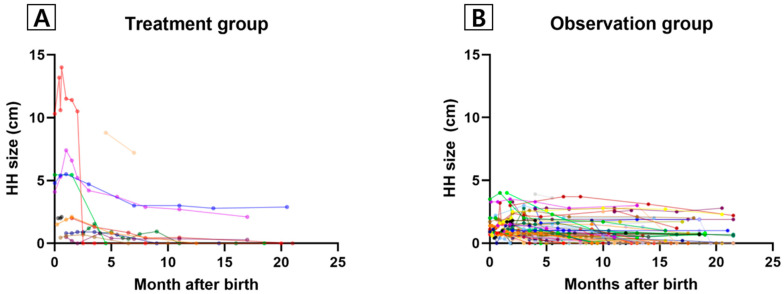
Trends in HH size during the first 2 years of life. (**A**) Treatment group. (**B**) Observation group. Abbreviations: HH, hepatic hemangioma.

The median (range) follow-up duration for all patients was 581 days (224–946 days, Table 1). The change in HH size in the treatment and observation groups during the first 2 years of life is shown in Figure 1. The maximum HH size in the observation group was 4 cm (Figure 1B). 

### 3.4. Trends in Serum AFP Levels and Half-Life in the First 2 Years of Life

**Figure 2:**
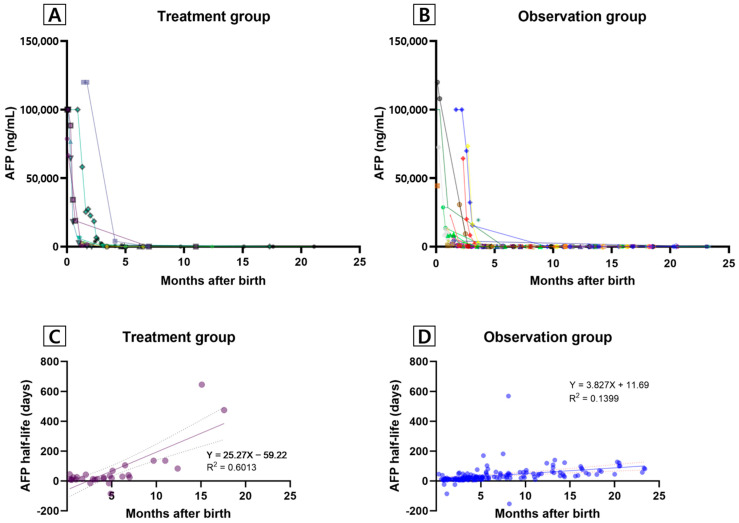
Serum AFP levels and half-life in the first 2 years of life. (**A**) Serum AFP levels in the treatment group. (**B**) Serum AFP levels in the observation group. (**C**) AFP half-life in the treatment group. (**D**) AFP half-life in the observation group. Abbreviations: AFP, alpha-fetoprotein.

Serum AFP levels were measured in 91.6% (11/12) of the patients in the treatment group and 77.3% (58/75) of those in the observation group. Serum AFP levels in both groups rapidly declined during the first 3 months of life and were almost undetectable after 6 months. The observation group exhibited a more gradual decreasing trend than the treatment group (Figure 2A,B). Wide individual variations in serum AFP levels were observed in both groups. A linear regression analysis of AFP half-life in the treatment group revealed a significant correlation between the serum AFP level and age after birth (treatment group: half-life = 25.57 × ‘months after birth’ − 59.22, R^2^ = 0.6013, *p* < 0.001, Figure 2C; observation group: half-life = 3.827 × ‘months after birth’ + 11.69, R^2^ = 0.1399, *p* < 0.001, Figure 2D). 

### 3.5. Treatment and Outcomes of Challenging Cases

Among the patients who underwent treatment, we summarized detailed treatment and outcome data of three patients with large HH who were successfully treated using a single therapeutic agent, step-by-step medical treatment, and surgical resection. 

**Figure 3:**
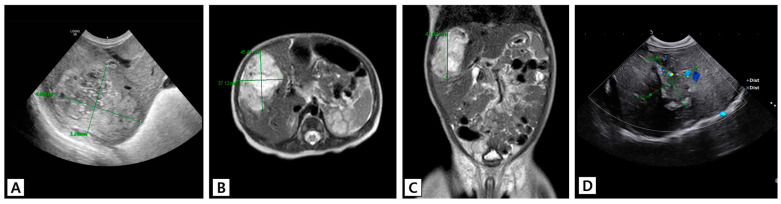
The US and MRI images of Patient 7, who had a large HH that partially resolved after treatment using a single therapeutic agent. (**A**) US performed on the day of birth shows a 4.8 × 3.3 cm sized mass on the right liver. The patient had mild feeding difficulty. (**B**,**C**) MRI performed on the ninth day after birth shows a large congenital hemangioma in the right lobe of the liver (S8 and S5) with internal necrosis and hemorrhage. Oral propranolol was initiated. (**D**) US performed at the age of 6 months shows that the largest diameter of the lesion decreased to 3 cm after treatment using oral propranolol. Abbreviations: HH, hepatic hemangioma; AFP, alpha-fetoprotein; US, ultrasound; MRI, magnetic resonance imaging.

The patient in Case 1 (Patient 7), a boy born at 37 weeks with a birth weight of 3270 g, had a 4.8 × 3.3 cm sized hepatic mass that was detected on US (Figure 3A). The patient was hemodynamically stable and only had mild feeding difficulty. HH was confirmed on abdominal MRI, which showed a 4.1 × 4.5 × 3.7 cm sized large, enhanced, heterogeneous mass in the right lobe of the liver (Figure 3B,C). Oral propranolol was started from the ninth day after birth because the diameter of the mass increased by 1 cm in a week. Serial ultrasound studies showed no further increase in the size of the HH, and the patient’s feeding difficulty was resolved. Imaging performed at the 6-month follow-up revealed that the largest diameter of the HH had reduced to 3 cm. The size of the mass remained stable over the next 2 years (Figure 3D).

**Figure 4:**
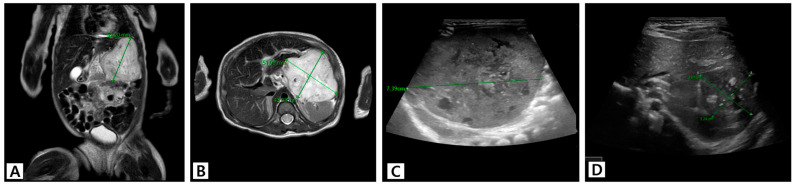
The US and MRI images of Patient 10, who had a large HH that partially resolved after step-by-step medical treatment. (**A**,**B**) MRI performed on the third day after birth shows an approximately 5.2 × 4.3 × 5.1 cm sized lobulated T2 hyperintense mass in the left lateral segment of the liver. The patient presented with feeding difficulty and abdominal distension. Oral propranolol was administered for 3 weeks from the 6th day of birth, and prednisolone was added at 1 month of age. (**C**) US performed at the age of 6 weeks revealed that the HH had increased in size up to 7.2 × 5.2 × 7.4 cm, with no improvement in symptoms. Oral sirolimus was initiated. (**D**) Follow-up US performed at 5 months of age shows that the tumor had decreased to 3.7 × 3.2 × 2.8 cm. Abbreviations: HH, hepatic hemangioma; US, ultrasound; MRI, magnetic resonance imaging.

The patient in Case 2 (Patient 10), a girl born at 39 weeks with a birth weight of 3.3 kg, was diagnosed with a 5.2 cm sized HH. Abdominal MRI performed on the third day after birth showed an approximately 5.2 × 4.3 × 5.1 cm sized lobulated T2 hyperintense mass on the left lateral segment of the liver (Figure 4A,B). The patient presented with recurrent vomiting and abdominal distension but no hemodynamic instability. Oral propranolol was administered for 3 weeks from the sixth day after birth. Prednisolone was added at 1 month of age. US performed at the age of 6 weeks revealed that the HH continued to increase in size up to 7.2 × 5.2 × 7.4 cm. In addition, the patient showed no improvement in symptoms; therefore, oral sirolimus was initiated (Figure 4C). The HH decreased slightly on the fifth day of sirolimus treatment, and the patient’s clinical symptoms began to improve from the tenth day of sirolimus treatment. Prednisolone was tapered out, and US performed at the age of 5 months showed that the tumor size had decreased to 3.7 × 3.2 × 2.8 cm (Figure 4D). Sirolimus was discontinued at 8 months of age. Thereafter, the HH gradually decreased in size, measuring 2.1 × 2.0 × 2.1 cm at 18 months of age.

**Figure 5:**
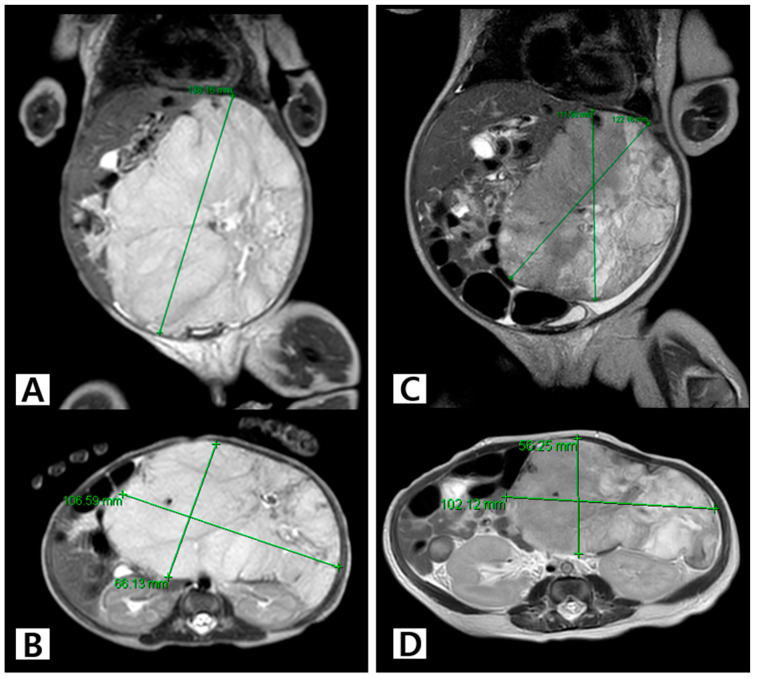
The MRI scans of Patient 11, who underwent step-by-step medical treatment and surgical resection, which led to a complete resolution of the tumor. (**A**,**B**) Coronal and axial MRI performed on the fifth day of life shows a 14 × 10 × 6.5 cm sized, well-defined, and lobulated mass suggestive of HH. The patient presented with abdominal distension, vomiting, and respiratory difficulty. (**C**,**D**) Coronal and axial MRI performed at the age of 6 weeks shows a partial reduction in the mass without clinical improvement in symptoms, after hepatic artery embolization and the administration of oral propranolol, prednisolone, and sirolimus. Abbreviations: HH, hepatic hemangioma; MRI, magnetic resonance imaging.

**Figure 6:**
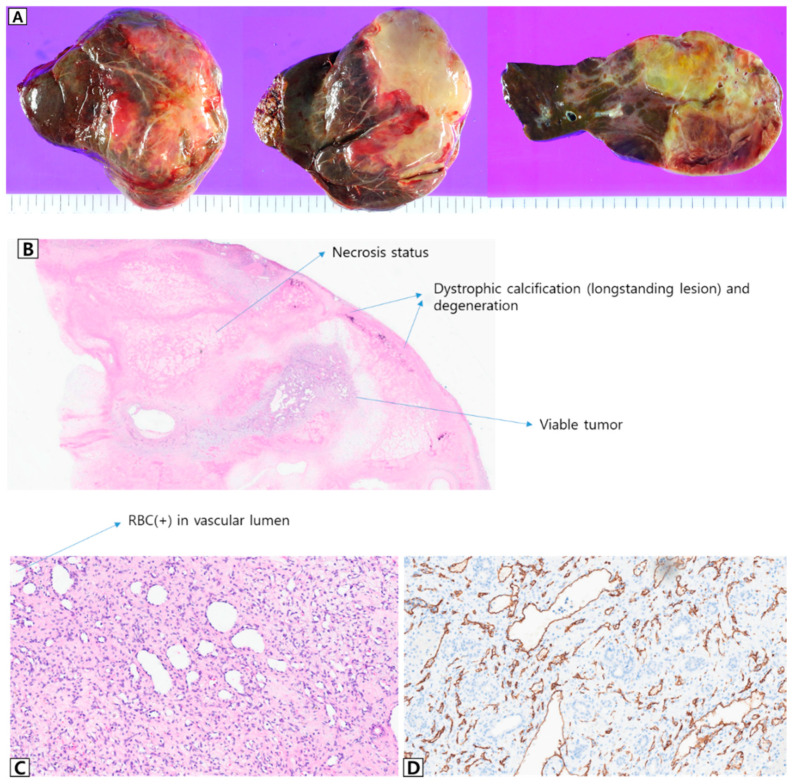
Postoperative pathology results of Patient 11, who underwent stepwise medical treatment and surgical resection. (**A**) Gross appearance of excised large hemangioma. A multi-lobular yellowish mass (largest diameter, 11.2 cm) with irregular fibrosis and myxoid degeneration was observed. The mass was abutting Glisson’s capsule and 3 cm away from the hepatic parenchymal resection margin. (**B**) Hematoxylin and eosin stain, scan view. The mass showed widespread internal necrosis and multifocal dystrophic calcification, with only a focal viable tumor remaining. (**C**) Hematoxylin and eosin stain, 15× magnified view. A viable portion of the tumor showed variable-sized (capillary- to arteriole-sized) vascular lumens with internal erythrocytes, lined with bland-looking flat endothelial cells. (**D**) CD34 immunohistochemical staining, 15× magnified view. The tumor cells showed diffuse immunoreactivity to CD34, confirming the endothelial differentiation of the tumor.

The patient in Case 3 (Patient 11), a boy with a birth weight of 3.3 kg, was born prematurely at 35 + 1 weeks for the diagnosis and management of a large hepatic mass. MRI performed at the age of 5 days showed a 14 × 10 × 6.5 cm sized, well-defined, and lobulated mass, suggestive of HH (Figure 5A,B). The patient presented with abdominal distension, vomiting, and respiratory difficulty. Oral propranolol was initiated on the seventh day after birth, and prednisolone was added from the thirteenth day; however, the patient showed no clinical improvement. Embolization performed at the age of 15 days resulted in only a temporary decrease in tumor size to approximately 12 × 10 × 5.5 cm on MRI (Figure 5C,D). The administration of sirolimus was ineffective and did not yield any improvement. Left lateral sectionectomy of the liver was performed at 2 months (corrected age, 1 month) when the patient began to show heart failure symptoms. The patient’s pathological signs were consistent with those of IH (Figure 6). However, all his clinical symptoms improved, and he was discharged without complications. 

## 4. Discussion

This study was a detailed analysis of the clinical presentation and prognosis of patients with HH using real-world data collected over 15 years. All the patients in the present study showed a rapid decrease in AFP levels during the first 3 months after birth, ruling out the possibility of malignancy. The patients in the observation group were asymptomatic at the diagnosis, and none of them progressed to being symptomatic. The patients in the treatment group showed various clinical presentations and treatment outcomes. Of note, two of seven patients with focal HH underwent surgical resection due to heart failure, whereas five patients achieved partial or complete resolution with medical treatment. None of the three patients with multifocal lesions had life-threatening symptoms, and the lesions completely resolved after medical treatment alone. Among the two patients with diffuse HH, one died from extensive heart failure and Kasabach–Merritt syndrome, whereas another asymptomatic patient showed a partial response to beta-blockers. Our data showed that the asymptomatic patients who had HHs with a diameter of 4 cm or less showed involution with close observation alone. 

HH is divided into CH and IH. CH is typically present in utero, fully formed at birth, and histologically Glut-1-negative, whereas IH develops after birth and is histologically Glut-1-positive. Intralesional calcification occurs almost exclusively in CH. The differentiation of CH from IH is essential because of their different natural courses in terms of mass involution, as well as the minimal possibility of the malignant transformation of IH. However, the precise differentiation of CH from IH is difficult because not all CH cases can be diagnosed on antenatal US, and HH lesions are generally not biopsied due to the risk of bleeding. In addition, the principles of treatment of CH and IH do not differ in terms of clinical symptoms and choice of treatment options. Therefore, the most important aspect of treating HH is proper management and regular follow-up, regardless of its histological type. The Hepatic Hemangioma Registry recommends that liver US be performed at progressively longer intervals after the diagnosis, with an initial 2-week interval, and the addition of 2 weeks to the interval after each stable evaluation [5]. In addition, follow-up should be maintained for at least 1 year and until US shows stable tumor size and vascularity. 

Large HH is typically defined as a mass >4 cm in diameter that can cause serious complications [9]. Without prompt intervention, neonatal mortality rates for HH range from 30 to 100% [10,11,12]. The prevalence of HH in a recent large-scale single-center study was 0.64/10,000 births [13]. In the present study, large HH was diagnosed in 5 of 87 (5.7%) patients. All the patients exhibited clinical symptoms and were treated without observation. The HH size in the observation group did not exceed 4 cm, and no further increase in size was observed after 1 year of follow-up. Notably, Rutten et al. reported a spontaneous evolution of HHs of various sizes, one of which had a size of 5.3 × 2.9 × 5.3 cm. 

HH must be distinguished from malignancies, such as hepatoblastoma, that may occur during the neonatal period [14]. Elevated AFP levels during the proliferative phase of IH have been reported in a few studies [15,16]. As infants have a wide range of normal AFP levels and show elevated AFP levels compared with adults [17], it is important to confirm a decrease in the AFP level during the follow-up period for patients with HH. In other words, a significantly elevated AFP level in newborns with hepatoblastoma is concerning [18]. Notably, neonates generally show highly elevated serum AFP levels (full-term neonates, 41,687 µg/L; preterm neonates, 158,125 µg/L) [19]. Wu et al. reported that the AFP half-life between birth and 2 weeks after birth was 5.5 days, that between 2 weeks and 2 months is 11 days, and that between 2 and 4 months is 33 days [17]. The data of the present study indicated that the treatment group showed higher initial and last follow-up AFP levels than the observation group. AFP half-life was more strongly correlated with age in the treatment group than in the observation group. However, the individual trends in the AFP level and half-life varied in both groups. Moreover, there was no correlation between initial HH size and the AFP level or half-life. However, the interpretation of this finding is limited by the different timepoints at which HH was diagnosed.

Oral beta-blockers (propranolol), first introduced in 2008, are currently considered the first-line therapy for HHs [20]. Vasoconstriction, endothelial cell apoptosis, and decreased angiogenesis have been proposed to explain the effects of propranolol on hemangiomas [21]. The time-to-response onset varies from 4.3 weeks to 8.7 weeks [22]. The poor response of large HHs to beta-blockers may be associated with arteriovenous shunting [23]. The effect of beta-blockers on IH seems to be related to the diameter of the hemangioma [24]. However, there is no suggested cut-off diameter for the indication of beta-blockers. In the present study, all the patients in the treatment group except for Patient 1 received oral propranolol. Notably, Patient 7, who had an approximately 5 cm mass and only mild feeding difficulty, responded to single-beta-blocker therapy after 3 months of administration; notably, a partial increase in size was observed during the first month of life. 

Sirolimus is a mammalian target of a rapamycin inhibitor, blocking downstream protein synthesis involved in the regulation of the cell cycle, and therefore vascular endothelial proliferation, resulting in antitumor and antiangiogenic effects [25]. It is used as an immunosuppressive, antiangiogenic, and cytostatic agent in clinical practice; however, its application in the treatment of vascular anomalies was only recently reported [26]. A recent systematic review of 73 studies that included 373 patients indicated that sirolimus showed promising results in the management of various vascular anomalies. In the present study, Patient 10 in the treatment group, who showed no response to beta-blockers and prednisolone, was successfully treated with sirolimus from 6 weeks to 8 months after birth without complications. In contrast, Patient 11, who had a large HH, ultimately required surgical resection as he did not respond to sirolimus, only exhibiting sepsis-like complications. The data of the present study and previous reports indicate that sirolimus could be an ideal option for rapidly growing hemodynamically stable HHs; however, its side effects should be closely monitored [27,28].

The surgical resection of HH is rarely performed, mainly only for lesions that are refractory to medical treatment and hemodynamically unstable [29]. Decision making regarding surgical treatment of HHs is a complex process that extends beyond considering the maximal diameter of the tumor alone. Clinical indicators, such as progressive abdominal symptoms, rupture risk, Kasabach–Merritt syndrome, and diagnostic ambiguity, are integral to formulating a surgical plan [30,31]. The efficacy and feasibility of standard treatments such as partial hepatectomy and enucleation have been well documented [32,33]. Nevertheless, the presence of sizable hemangiomas increases the surgical challenge [34]. These lesions can exert a compressive effect on adjacent vessels, complicating meticulous ligation for preventing vascular inflow to tumors and potentially distorting anatomical landmarks such as the vena cava, thereby elevating the risk of intraoperative bleeding. Furthermore, as these physical constraints are more critical in infants, employing minimally invasive techniques for the resection of extensive masses remains a challenge [35,36]. Thus, careful preoperative mapping of the vascular supply using angio-CT or angiography is essential. Such measures are vital for reducing intraoperative complications and accurately assessing the remnant liver volume [37].

Of the 11 patients in the treatment group in the present study, 2 underwent surgical resection at 41 and 61 days. None of the two patients responded to medical treatment for over 1 month; in addition, they showed heart failure symptoms before surgery. Patient 11 had a considerably large HH with a diameter of 14 cm. Medical treatment using propranolol, steroids, and embolization induced temporary partial reduction in the size of the mass and helped delay the time of surgery. In contrast, the administration of sirolimus had no effect on mass reduction and only caused suspicious sepsis-like complications. Moreover, the patient showed delayed postoperative wound healing, which may have been influenced by the preoperative administration of sirolimus. Considering the adverse effects of such pharmacological treatments, rigorous monitoring of wound healing after HH resection is essential [38].

This study has some limitations. First, this is a retrospective and single-center study, so the possibility of selection bias cannot be ruled out. Second, CH and IH were not differentiated in this study. Third, the choice and duration of medical treatment were determined by each physician, and not based on a common protocol. Nevertheless, this study is valuable in that it provided comprehensive clinical information regarding changes in tumor size and the trend in AFP levels and half-life in patients with HH according to the treatment administered, as well as detailed progress of each patient. However, the results of this study should be interpreted cautiously because of the limited number of patients included in this study, and the fact that tumor size and the timing of AFP measurement varied between patients.

In conclusion, we conducted this study to analyze the clinical courses of patients with HH based on our experience of over 15 years in a tertiary single-center institution. Our findings obtained over 1 year of serial follow-up of each patient and the detailed clinical courses of the challenging cases may help physicians in decision making regarding HH treatment. Moreover, this study demonstrated that patients with large HHs and mild symptoms can be treated using stepwise pharmacological therapy, including beta-blockers, steroids, and sirolimus, whereas more aggressive surgical treatment of tumors that do not respond to initial pharmacological therapy may help shorten the treatment period and improve patient outcomes. 

## Figures and Tables

**Table 1:** Demographic characteristics and clinical courses of the study population.

	Total (*n* = 87)	Treatment Group (*n* = 12)	Observation Group (*n* = 75)	*p*
Male, *n* (%)	43 (49.4)	6 (50.0)	37 (49.3)	0.966
Gestational age, weeks	36.4 (32.0–38.2)	35.4 (32.6–39.0)	36.7 (31.8–38.2)	0.873
Birth weight, kg	2.59 (1.72–3.26)	3.27 (1.87–3.48)	2.58 (1.70–3.15)	0.193
Prenatal diagnosis of HH, *n* (%)	13 (14.9)	4 (33.3)	9 (12.0)	0.076
Age at diagnosis of HH, days	61.0 (21.0–126.0)	15.5 (1.0–37.8)	75 (27–137)	0.002
Diagnosis method, *n* (%)				0.001
Only US	45 (51.7)	1 (8.3)	44 (58.7)	
US + MRI or CT	42 (48.3)	11 (91.7)	31 (41.3)	
Appearance of HH, *n* (%)				0.027
Focal	61 (70.1)	7 (58.3)	54 (72)	
Multifocal	24 (27.6)	3 (25.0)	21 (28)	
Diffuse	2 (2.3)	2 (16.7)	0 (0)	
Heart failure, *n* (%)	5 (5.7)	3 (25.0)	2 (2.7)	0.018
HH size, cm				
Size at initial diagnosis	1.0 (0.4–10.3)	2.2 (0.5–10.3)	1.0 (0.4–4.0)	0.035
Maximum size	1.1 (0.4–13.2)	2.1 (0.7–13.2)	1.1 (0.4–4.0)	0.007
Size at last follow-up	0 (0–7.2)	0 (0–7.2)	0 (0–0.3.6)	0.780
Serum AFP level, initial, ng/mL	939.7 (3.9–120,000)	76,818.7 (231.2–120,000)	627.2 (3.93–120,000)	0.001
Serum AFP level, last, ng/mL	10.4 (1.3–76,818)	98.4 (4.3–76,818.7)	8.7 (1.3–44,439)	0.026
Treatment outcomes, *n* (%)				1.000
Complete resolution	53 (60.9)	8 (66.7)	45 (60)	
Partial resolution	24 (27.6)	3 (25.0)	21 (28)	
No resolution	10 (11.5)	1 (8.3)	9 (12.0)	
Follow-up duration, days	581 (224–946)	631.5 (152.0–912.3)	570.0 (224.0–963.0)	0.622

Footnotes: Data are presented as numbers (% of the same column) or medians (range). Abbreviations: HH, hepatic hemangioma; US, ultrasound; MRI, magnetic resonance imaging; CT, computed tomography; AFP, alpha-fetoprotein.

**Table 2:** Treatment and clinical outcomes of patients with hepatic hemangiomas.

No.	Sex	Prenatal Diagnosis	Age at Diagnosis (days)	GA (wks)	BW (gm)	Associated Anomaly	Appearance	Initial/ Maximum Size (cm) *	Symptoms	Indication of Treatment **	Medical Treatment (Days after Birth)	Embolization (Days after Birth)	Operation (Days after Birth)	Outcomes /Time to Complete Resolution (days)	Complications
1	M	Yes	1	34	1990	None	Focal	5.46/5.46	CHF	1	PL (4–5) +INFa (4–41)	Yes (2)	Yes, Right hemihepat-ectomy (41)	Complete/41 (at op day)	Postoperative cholestasis, delayed surgical wound healing
2	F	No	38	26	960	ASD	Multifocal	0.5/0.8	Cutaneous hemangiomas	4	BB (147–326)	No	No	Complete/844	None
3	F	No	10	40	3800	ASD	Diffuse	2/2.1	KMS, CHF, hypothyroidism	3	BB (11–18) +PL (11–18)	No	No	No	Pulmonary hemorrhage, death
4	F	No	29	40	3530	Biliary atresia	Focal	0.8/0.9	N	2	BB (63–157)	No	No	Complete/115	None
5	F	No	6	34	1830	ASD	Focal	1.5/2.1	N	2	BB (45–150)	No	No	Partial	None
6	M	No	37	38	2500	None	Multifocal	2/2	Cutaneous hemangiomas	4	BB (40–194)	No	No	Complete/666	None
7	M	Yes	0	37	3270	ASD	Focal	4.8/5.8	Feeding difficulty	2	BB (11–379)	No	No	Partial	None
8	M	No	21	32	1530	ASD	Multifocal	0.46/0.7	Cutaneous hemangiomas	4	BB (36–295)	No	No	Complete/180	None
9	M	No	136	37	3260	None	Focal	8.8/8.8	Asymptomatic	2	BB (148–221)	No	No	Partial	None
10	F	Yes	1	39	3320	None	Focal	5.2/7.4	Abdominal distension, feeding difficulty	1	BB (3–200) +PL (28–82) +SRL (37–200)	No	No	Partial	None
11	M	Yes	0	35	3300	VSD, ASD, syringomyelia	Focal	13.8/14	Respiratory distress, abdominal distension, jaundice, CHF, hypothyroidism	1	BB (6–60) +PL (13–57) +SRL (30–37)	Yes (15)	Yes, Left lateral sectionectomy of liver (61)	Complete/61 (at op day)	Septic shock after SRL, delayed surgical wound healing
12	F	No	68	35	2270	Beckwith–Wiedemann syndrome, ASD, cleft palate, ileal atresia	Diffuse	0.82/1.54	N	2	BB (93–233)	No	No	Partial	None

Abbreviations: M, male; F, female; GA, gestational age; BW, birth weight; CHF, congestive heart failure; KMS, Kasabach–Merritt syndrome; PL, prednisolone; BB, beta-blocker; INFa, interferon alpha; SRL, sirolimus; ASD, atrial septum defect; VSD, ventricular septum defect. * Tumor size is defined as the longest diameter of the largest tumor. ** The indication of treatment was classified into four categories: (1) large HH that induced life-threatening symptoms, (2) HHs that increased in number and size such that spontaneous regression was not expected, (3) diffuse HHs accompanied by hepatic failure, (4) other involvement of other organs including the skin.

## Data Availability

The data presented in this study are available on request from the corresponding author due to ethical reasons.
